# Two Cases of Double Spontaneous Dislocation of the Lens

**Published:** 1870-01

**Authors:** Henry W. Williams

**Affiliations:** Boston, President of the American Ophthalmological Society


### Miscellanous.

### Two Cases of Double Spontaneous Dislocation of the Lens.

By HENRY W. WILLIAMS, A. M., M. D., Boston, President of the American Ophthalmological Society.

On July 13th, 1869, I saw Mr. M., a healthy man of 35, who gave me the following history:—

At about 18 years of age he was told that his iris quivered in both eyes; and this he had himself been able to observe on looking in a mirror. Probably the suspensory attachments of the crystalline were even then relaxed or separated to a greater or less extent.

About a year and a half since, the lens of the right eye passed through the pupil into the anterior chamber, and this phenomenon has been repeated, at intervals varying from two or three weeks to as many month; always occurring when he stooped or made an effort in lifting. Usually, he has been able to replace the lens by shutting himself for a few moments in a dark closet. The displacement has not generally been accompanied with pain.

He has noticed that when he leaned his head towards the right he saw better, when towards the left he scarcely saw at all with this eye. He wears No. 6 concave glasses, aad with these reads No. xxx. of test types at ten feet distance with his left eye. Vision good for reading with this eye.

Four days before I saw him, the right lens became displaced into the anterior chamber, and has remained there, giving him a dull pain in the globe, and causing very slight injection. It appears like a large drop of oil between the cornea and the iris. The pupil is considerably dilated, and the iris is distended by the over-lying lens.

He was placed upon his back in a dark room, and the cornea was gently rubbed with the upper lid, until, after a few minutes, the lens slipped back into the posterior chamber. When his head was upright the lens now covered the inner half of the field of the pupil; when the head was leaned towards the right shoulder nearly the whole field of the much dilated pupil was covered by the lens; but when it leaned towards the left shoulder the margin only of the lens was visible at the inner edge of the pupil. Evidently the displacement was lateral, without any tilting backwards.

When the lens was nearest its normal position he saw, with his No. 6 *concave* glass, nearly as well as with the other eye; when it was laterally displaced he saw best with No. 6 *convex*.

On examination with the ophthalmoscope the fundus of both eyes seemed normal. The iris was tremulous in the left eye, and there can be no doubt that in this eye also the attachments of the suspensory ligament have given way to some extent; but as the pupil in this eye was contracted, as a consequence of the large dilatation in the right eye, it was not possible to determine how much dislocation of the lens existed unless after artificial enlargement of the pupil, to which it was not desirable to resort lest anterior displacement might perhaps occur in this eye.

Three large doses of extract of calabar bean were put into the right eye; producing at the end of an hour, an evident though but slight diminution in the size of the pupil. This proved, however, insufficient to prevent the lens from again falling into the anterior chamber on his making a slight movement forward. It was again replaced, by friction upon the cornea, and he was kept on his back, but not in the dark, for some time longer, But even the slight effort of rising from his chair, without any perceptible forward movement of the head, sufficed to again propel the lens through the pupil. It is not unlikely that some accommodative effort of the eye may have had an agency in the displacement.

Replacement being once more effected, he was dismissed with directions to put a square of calabar gelatine into the eye every hour, in the hope that sufficient contraction of the pupil might be induced to form a permanent barrier to the escape of the lens into the anterior chamber.

The next day the friend who accompanied him reported to me that the lens had been several times displaced. He was advised to remain quiet during the day and to continue the calabar, and if necessary, to report to me the next morning before setting out for his home.

He was also cautioned that he must avoid sudden exertion, lifting, &c.; since he might thus dislocate one or both lenses.

Nov. 11th, 1869.—A boy of about 12 years of age was brought to the Ophthalmic Clinique at the City Hospital, both of whose eyes exhibited partial lateral displacement and slight opacity of the crystalline. His mother was sure he had not good vision; but its exact amount could not readily be ascertained on account of the mental imbecility of the patient.—*Boston Journal, Dec.*, 1869.

